# SHBG, Free Testosterone, and Type 2 Diabetes Risk in Middle-aged African Men: A Longitudinal Study

**DOI:** 10.1210/jendso/bvae129

**Published:** 2024-07-19

**Authors:** Ikanyeng D Seipone, Amy E Mendham, Karl-Heinz Storbeck, Imken Oestlund, Clement N Kufe, Tinashe Chikowore, Maphoko Masemola, Nigel J Crowther, Andre Pascal Kengne, Shane Norris, Tommy Olsson, Todd Brown, Lisa K Micklesfield, Julia H Goedecke

**Affiliations:** Biomedical Research Innovation Platform, South African Medical Research Council, Cape Town 7505, South Africa; Riverland Academy of Clinical Excellence, Riverland Mallee Coorong Local Health Network, South Australia Health, Berri, SA 5343, Australiacountry; South African Medical Research Council/WITS Developmental Pathways for Health Research Unit, Department of Paediatrics, School of Clinical Medicine, Faculty of Health Sciences, University of the Witwatersrand, Johannesburg 2000, South Africa; Health through Physical Activity, Lifestyle and Sport Research Centre, FIMS International Collaborating Centre of Sports Medicine, Division of Physiological Sciences, Department of Human Biology, Faculty of Health Sciences, University of Cape Town, Cape Town 7701, South Africa; Department of Biochemistry, Stellenbosch University, Stellenbosch 7602, South Africa; Department of Biochemistry, Stellenbosch University, Stellenbosch 7602, South Africa; South African Medical Research Council/WITS Developmental Pathways for Health Research Unit, Department of Paediatrics, School of Clinical Medicine, Faculty of Health Sciences, University of the Witwatersrand, Johannesburg 2000, South Africa; South African Medical Research Council/WITS Developmental Pathways for Health Research Unit, Department of Paediatrics, School of Clinical Medicine, Faculty of Health Sciences, University of the Witwatersrand, Johannesburg 2000, South Africa; South African Medical Research Council/WITS Developmental Pathways for Health Research Unit, Department of Paediatrics, School of Clinical Medicine, Faculty of Health Sciences, University of the Witwatersrand, Johannesburg 2000, South Africa; Department of Chemical Pathology, National Health Laboratory Service and University of the Witwatersrand Faculty of Health Sciences, Johannesburg 2000, South Africa; Non-Communicable Diseases Research Unit, South African Medical Research Council, Cape Town 7505, South Africa; South African Medical Research Council/WITS Developmental Pathways for Health Research Unit, Department of Paediatrics, School of Clinical Medicine, Faculty of Health Sciences, University of the Witwatersrand, Johannesburg 2000, South Africa; Department of Public Health and Clinical Medicine, Umeå University, Umeå 90187, Sweden; Division of Endocrinology and Metabolism, Johns Hopkins University School of Medicine, Baltimore, MD 21218, USA; South African Medical Research Council/WITS Developmental Pathways for Health Research Unit, Department of Paediatrics, School of Clinical Medicine, Faculty of Health Sciences, University of the Witwatersrand, Johannesburg 2000, South Africa; Biomedical Research Innovation Platform, South African Medical Research Council, Cape Town 7505, South Africa; South African Medical Research Council/WITS Developmental Pathways for Health Research Unit, Department of Paediatrics, School of Clinical Medicine, Faculty of Health Sciences, University of the Witwatersrand, Johannesburg 2000, South Africa

**Keywords:** free testosterone, sex hormone-binding globulin, dysglycaemia, type 2 diabetes, Africa

## Abstract

**Objectives:**

To investigate longitudinal changes in SHBG and free testosterone (free T) levels among Black middle-aged African men, with and without coexistent HIV, and explore associations with incident dysglycaemia and measures of glucose metabolism.

**Design:**

This longitudinal study enrolled 407 Black South African middle-aged men, comprising primarily 322 men living without HIV (MLWOH) and 85 men living with HIV (MLWH), with normal fasting glucose at enrollment. Follow-up assessments were conducted after 3.1 ± 1.5 years.

**Methods:**

At baseline and follow-up, SHBG, albumin, and total testosterone were measured and free T was calculated. An oral glucose tolerance test at follow-up determined dysglycaemia (impaired fasting glucose, impaired glucose tolerance, type 2 diabetes) and glucose metabolism parameters including insulin sensitivity (Matsuda index), insulin resistance (homeostasis model assessment of insulin resistance), and beta(β)-cell function (disposition index). The primary analysis focussed on MLWOH, with a subanalysis on MLWH to explore whether associations in MLWOH differed from MLWH.

**Results:**

The prevalence of dysglycaemia at follow-up was 17% (n = 55) in MLWOH. Higher baseline SHBG was associated with a lower risk of incident dysglycaemia (odds ratio 0.966; 95% confidence interval 0.945-0.987) and positively associated with insulin sensitivity (β = 0.124, *P* < .001) and β-cell function (β = 0.194, *P* = .001) at follow-up. Free T did not predict dysglycaemia. In MLWH, dysglycaemia prevalence at follow-up was 12% (n = 10). Neither baseline SHBG nor free T were associated with incident dysglycaemia and glucose metabolism parameters in MLWH.

**Conclusion:**

SHBG levels predict the development of dysglycaemia in middle-aged African men but do not exhibit the same predictive value in MLWH.

Reduced circulating testosterone is one of the risk factors for insulin resistance and type 2 diabetes (T2D) in men [1-5]. With advancing age, both total testosterone (total T) and, to a greater extent, free testosterone (free T) decrease [6, 7]. In contrast, SHBG, the glycoprotein that transports testosterone and regulates its bioavailability increases with healthy aging [6, 8-10]. Notably, recent data suggest that low SHBG is associated with impaired glucose metabolism independent of testosterone and, accordingly, has been identified as one of the factors that play a role in the pathogenesis of T2D [1, 11-13]. Importantly, the relationship of SHBG and free T with T2D has been sparsely studied in sub-Saharan Africa (SSA), with scanty cross-sectional data showing high levels of SHBG in men living with T2D in Nigeria [14] and South Africa [15] and low levels of free T in men living with T2D from Ghana [16], Nigeria [17], and South Africa [15, 18]. However, it is not clear if alterations in testosterone or SHBG predict T2D in Black African men. This is especially pertinent, as SSA has the highest predicted relative rate of increase in T2D [19] and presents with a different T2D pathogenesis compared to White European counterparts [20].

Sub-Saharan Africa not only has challenges with an increasing burden of T2D, but it is also the world's epicenter of HIV infections, accounting for more than 70% of the infections, with the numbers of those >50 years living with HIV estimated to triple by 2040 [21-23]. While earlier studies indicate a higher likelihood of impaired glucose metabolism among people living with HIV compared to those without HIV [24-26], more recent studies from SSA report inconsistent findings, particularly in the context of newer antiretroviral therapies (ART) [27, 28]. Some studies have reported increased risk of diabetes [27, 28] and others no association of HIV and ART and prevalent diabetes [29-31]. Notably, studies in the United States have reported that hypogonadism is common among men living with HIV (MLWH) despite effective ART [1, 3]. However, data from SSA on the prevalence of hypogonadism in those living with HIV is scarce. Accordingly, the aims of the study were to investigate longitudinal changes in SHBG and free T levels among Black middle-aged African men, with and without co-existent HIV, and explore associations with incident dysglycaemia and measures of glucose metabolism.

We hypothesized that (1) SHBG and/or free T levels will predict the development of dysglycaemia in middle-aged African men and (2) in a cohort of the Black-middle-aged MLWH, alterations of SHBG and/or free T levels with HIV will be linked to the development of dysglycaemia.

## Materials and Methods

### Study Population

**Figure 1.**
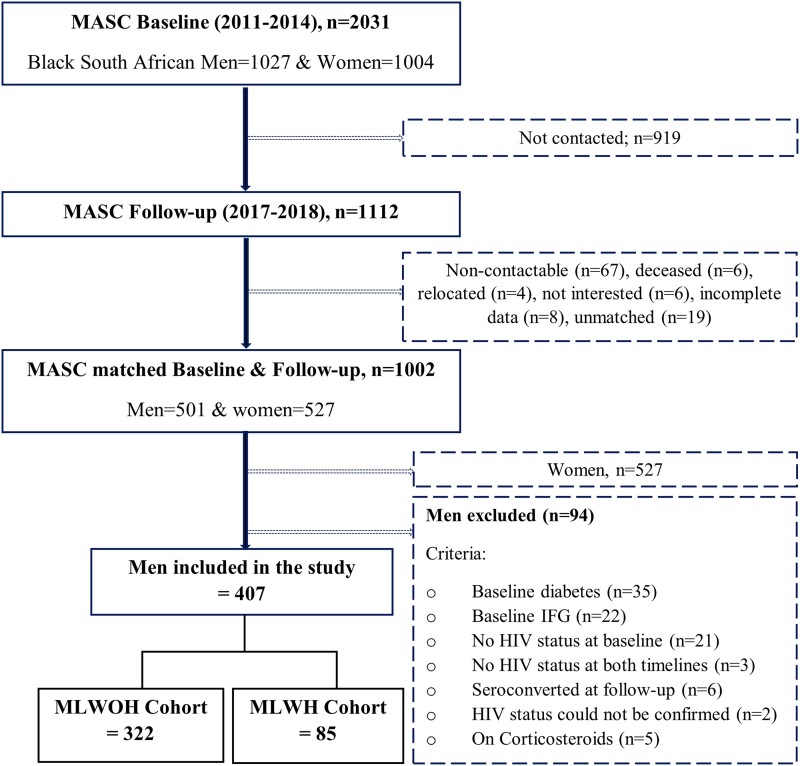
Description of the Middle-Aged Soweto Cohort longitudinal study. Abbreviations: MLWOH, men living without HIV; MLWH, men living with HIV.

This longitudinal study of men from the Middle-Aged Soweto Cohort was designed to investigate the determinants of T2D risk in Black South Africans. Baseline data was collected between 2011 and 2014 as part of the Africa Wits-INDEPTH partnerships for Genomic Research study and included 2031 participants (men = 1027 and women = 1004) [32]. Follow-up data on a conventional subsample (n = 1112) was collected between 2017 and 2018, as previously described [33]. This study included only men from this cohort. Of the 501 men, only those with normal fasting glucose from a standard oral glucose tolerance test (OGTT) at baseline and had follow-up data were included in the analysis (n = 407), as described in Fig. 1. The main analysis was performed on 322 MLWOH and a subanalysis on 85 MLWH to determine if significant associations in the MLWOH cohort differed from MLWH (Fig. 1).

The study was conducted in accordance with the Declaration of Helsinki and was approved by the University of the Witwatersrand Human Research Ethics Committee (reference no. M160604 and M160975). Prior to commencing the study, informed consent was obtained from participants after providing them with information about the study, procedures, and possible risks.

### Sociodemographics and HIV Status

A standardized questionnaire was used to collect data at both baseline and follow-up. The data included age; marital status; smoking (current smoker/nonsmoker); alcohol intake (consumes/does not consume); employment status (currently employed/not employed); education level (completed >12 years, 10-12 years, or ≤9 years of education); medical history; and chronic medication use (yes/no). HIV testing was completed using the rapid HIV test (One Step HIV ½ Whole Blood/Serum/Plasma Test: Wondfo Biotech, Co., Ltd., Guangzhou, China) to detect HIV antibodies in participants’ blood samples. Pre- and post-HIV test counseling was carried out, and participants with positive test results were referred to a clinic for appropriate management. Those with a confirmed HIV status at entry were asked to present their medication to the interviewer to record ART use.

### Body Composition Measurements

Whole body fat mass and regional body fat were measured at baseline and follow-up using dual-energy X-ray absorptiometry (Hologic Discovery A, Hologic Inc., Bedford, MA, USA) and analyzed with APEX software version 13.4.2.3. Fat mass (kg) represented the subtotal mass (total minus head). Visceral adipose tissue (VAT) was estimated from dual-energy X-ray absorptiometry as previously described [34]. Height and weight were measured at baseline and follow-up using a wall–mounted stadiometer (Holtain, Wales, UK) and a calibrated standard scale (TBF-410, Tanita Corporation, Arlington Heights, IL, USA), respectively. Fat mass index (FMI) was calculated by dividing subtotal fat mass (kg) by height^2^ (m^2^) and body mass index (BMI) as body weight (kg) divided by height^2^ (m^2^).

### Biochemical Measurements at Baseline and Follow-up

Fasting blood (10-12 hours) was drawn at 08:00 hours in serum-separating tubes tubes at both baseline and follow-up for the measurement of total T, SHBG, and albumin. The following measurements were conducted simultaneously for MLWH and MLWOH. SHBG was measured using the Abbott ARCHITECT Chemiluminescent Microparticle Immunoassay assay [Abbott cat. no. 8K26 (also 08K2625), RRID: AB_2895255, Abbott Laboratories, Barcelona, Spain] according to the manufacturer's instructions. Total T was measured using ultra-high performance liquid chromatography-tandem mass spectrometry as previously described [35, 36], and albumin was measured using the Bromocresol Green albumin assay kit (Sigma-Aldrich, St. Louis, MO, USA) and analyzed on a Roche Cobas C600 (Roche Diagnostics Corporation, Indianapolis, IN, USA). Free T was calculated from the measured total T, SHBG, and albumin using the Vermeulen equation [37].

### OGTT at Follow-up

At follow-up, an OGTT was completed to assess glucose tolerance and determine various glucose metabolism parameters. A fasting (10-12 hours) blood sample was drawn at 08:00 hours for the determination of plasma glucose and serum insulin and C-peptide concentrations. Thereafter, participants ingested 75 g of glucose dissolved in water, and 5 mL blood samples were collected at 30-minute intervals for 2 hours. Plasma glucose concentrations were measured using the Randox RX Daytona Chemistry Analyser (Randox Laboratories Ltd., London, UK), and serum insulin and C-peptide concentrations were measured using the Immulite® 1000 Immunoassay System (Siemens cat. no. LKIN1, RRID: AB_2750939 and Siemens cat. no. LKPEP1, RRID: AB_2757817, respectively, Siemens Chemiluminescent Healthcare GmbH, Henkestr, Germany).

Participants were classified as having normal glucose tolerance (fasting glucose <6.1 and 2-hour glucose <7.8 mmol/L), impaired fasting glucose (fasting glucose 6.1-6.9 mmol/L) impaired glucose tolerance (2-hour glucose 7.8-11.0 mmol/L), or T2D (fasting glucose >7.0 and/or 2-hour glucose ≥11.1 mmol/L and/or on diabetes medication) according to the World Health Organization guidelines [38]. Participants with impaired fasting glucose, impaired glucose tolerance, and T2D were combined and referred to as having dysglycaemia.

Measurements from the OGTT were used to estimate first-phase insulin response (insulinogenic index) [39], insulin secretion (C-peptide index) [39], insulin sensitivity (Matsuda index) [40], and insulin resistance [homeostasis model assessment of insulin resistance (HOMA-IR)] [41]. The β-cell function, assessed as the insulin response for a given level of insulin sensitivity, was estimated using the disposition index (DI) [42-44].

### Statistical Analysis

Data was analyzed using Statistical Package for Social Sciences software version 28 (IBM SPSS Statistics 28.0.0) and Stata/SE 18.0 (Stata Corporation). Data for each variable was assessed for normality using the Shapiro–Wilk's normality test. Continuous variables are presented as mean ± SD when normally distributed, median (25-75th percentile) when skewed, and count (%) for categorical data. Follow-up time was defined as the period from the baseline enrollment date to the date of the OGTT. Changes in body composition parameters over time were analyzed using quantile mixed regression models adjusted for baseline age and follow-up time. Binary logistic models are reported as odds ratio (OR) [95% confidence interval (CI)], and quantile regression models are reported as β (*P*-value). Separate analyses using the models described here were conducted in MLWOH and MLWH. The only distinction was in MLWH an additional covariate of antiretrovirals use was included in model 2.

#### Longitudinal analysis of SHBG and testosterone levels

Quantile mixed regression models were used to explore changes in SHBG and testosterone over time. The first minimally adjusted model, model 1, consisted of time, baseline age, and follow-up time. Model 2 included model 1 variables, as well as smoking and chronic medication use (hypertension, dyslipidemia, and/or diabetes) as well as total and central adiposity (FMI and VAT area).

#### Relationship of baseline SHBG and free T with dysglycaemia and glucose metabolism parameters at follow-up

Binary logistic regression models were performed to assess the relationship of SHBG and free T at baseline with dysglycaemia (yes/no) at follow-up. Model 1 consisted of baseline age and follow-up time. Model 2 included covariates as previously described.

The relationship between SHBG and free T with parameters of glucose metabolism was modeled using quantile regression. The model included the glucose metabolism parameters at follow-up as the outcome and SHBG and free T (baseline) as the independent variable. Models 1 and 2 included covariates as previously described.

Additional analysis were carried out to compare baseline sociodemographics and sex hormones between MLWOH and MLWH using *t*-tests, chi-square tests, and equivalents. Differences between the cohorts at baseline (baseline age, FMI, VAT, and smoking status) were included as covariates in regression models to assess SHBG and testosterone level differences between the 2 groups.

## Results

### Participant Characteristics and SHBG and Testosterone Levels Over Time in MLWOH

The median age at baseline was 50.0 [interquartile range] [45.0-56.0] years, BMI was 25.5 [21.4-29.5] kg/m^2^, and FMI was 6.39 [4.32-8.50] kg/m^2^ (Table 1) in MLWOH. The majority (70.2%) consumed alcohol, whereas only 48.3% of the men smoked cigarettes, and 54.7% were employed.

**Table 1.** Baseline and follow-up characteristics of middle-aged Black SA MLWOH

Variable	Baseline	Follow-up	*P*-value
n (%)	322	322	**—**
Age [IQR] years	50.0 [45.0-56.0]	53.0 [48.0-59.0]	**<.001**
Body composition*^a^*			
Height (m)	1.71 ± 0.06	—	—
Weight (kg)	74.1 [63.4-86.4]	74.5 [62.9-87.0]	.713
Body mass index (kg/m^2^)	25.5 [21.4-29.5]	25.7 [21.3-30.0]	.718
Fat mass (kg)	18.5 [12.7-24.8]	18.9 [12.5-24.1]	.643
Fat mass index (kg/m^2^)	6.39 [4.32-8.50]	6.41 [4.24-8.26]	.886
Visceral adipose tissue (cm^2^)	76.1[52.6-112.6]	79.7 [50.1-114.5]	.604
Hormones*^a^*			
SHBG (nmol/L)	38.7 [30.2-52.8]	40.0 [31.7-52.6]	.586
Total testosterone (nmol/L)	14.5 [11.0-18.7]	14.9 [11.1-18.6]	.621
Free testosterone (nmol/L)	0.26 [0.21-0.32]	0.26 [0.21-0.32]	.526

Skewed data reported as median (25th-75th percentile) and normally distributed reported as mean ± SD. Significant *P*-values are shown in bold.

Abbreviations: IQR, interquartile range; MLWOH, men living without HIV; SA, South African.

^*a*^Adjusted for baseline age and follow-up time.

Body composition parameters did not change over the 3.1 ± 1.5 year follow-up period before and after adjusting for baseline age and follow-up period (Table 1).

Baseline median concentrations of SHBG, total T, and free T of the MLWOH are presented in Table 1. SHBG concentrations did not change significantly over time (Table 1). This persisted after adjustments for the putative confounding variables (data not shown). Similarly, total T and free T did not change significantly over the 3.1 ± 1.5 years even after adjusting for putative confounders in model 2 (Table 1 and data not shown).

### Participant Characteristics and SHBG and Testosterone Levels Over Time in MLWH

The baseline age of MLWH was (47.0 [44.0-52.0]) years (Table 2). The majority (76.5%) of men smoked cigarettes and consumed alcohol (68.6%), and 54.1% were employed. Of the 85 MLWH, 69 (81%) were on ART, with 58 (84%) being treated with the non-nucleoside reverse transcriptase inhibitors regimen, 7 (10%) treated with protease inhibitors (PIs), and in 4 (6%) the specific ART was unknown.

**Table 2.** Baseline and follow-up characteristics of the middle-aged black SA MLWH

Variable	Baseline	Follow-up	*P*-value
n (%)	85	85	**—**
Age [IQR] years	47.0 [44.0-52.0]	50.0 [47.0-55.0]	**<.001**
Body composition*^a^*			
Height (m)	1.71 ± 0.06	—	—
Weight (kg)	62.0 [56.0-72.8]	60.8 [54.4-72.8]	.959
Body mass index (kg/m^2^)	21.3 [18.9-24.6]	21.1 [18.5-25.3]	.646
Fat mass (kg)	11.4 [9.11-20.0]	12.3 [8.63-17.4]	.444
Fat mass index (kg/m^2^)	4.08 [3.04-6.87]	4.28 [2.94-6.00]	.428
Visceral adipose tissue (cm^2^)	60.0 [41.4-98.5]	55.1 [41.7-82.3]	.591
Hormones*^a^*			
SHBG (nmol/L)	52.4 [37.8-74.9]	67.2 [46.2-100.7]	.**006**
Total testosterone (nmol/L)	17.9 [15.0-24.3]	20.3 [14.8-25.1]	.108
Free testosterone (nmol/L)	0.29 [0.22-0.34]	0.25 [0.20-0.32]	.**022**

Skewed data reported as median (25th-75th percentile) and normally distributed reported as mean ± SD. Significant *P*-values are shown in bold.

Abbreviations: IQR, interquartile range; MLWOH, men living without HIV; SA, South African.

^*a*^Adjusted for baseline age and follow-up time.

The body composition parameters did not change with time before and after adjusting for baseline age and follow-up period (Table 2).

When compared to MLWOH, MLWH were younger (*P* = .001) and had a lower BMI (*P* < .001), FMI (*P* < .001), and VAT (*P* = .012). MLWH were more likely to be current smokers (*P* < .001) than MLWOH, but the proportion of men consuming alcohol did not differ between groups (*P* = .229) (Supplementary Table 1 [45]).

Baseline and follow-up concentrations of SHBG, total T, and free T of MLWH are presented in Table 3. SHBG concentrations increased significantly in MLWH over the 3-year follow-up period (*P* = .006), with an absolute increase of 2.98 [−0.17 to 8.40] nmol/L per year and an annual relative change of 4.89 [-0.15 to 14.36] %. This increase was significant after adjusting for possible confounding effects of chronic medication use and smoking (*P* = .007), but it was no longer significant when adjusted for baseline total and central adiposity (FMI and VAT) (*P* = .320). Even though total T did not change with time (Table 2), free T decreased with time in MLWH in all models (Table 2, model 2; *P* = .05). The absolute decrease in free T was (−0.010 [−0.031 to 0.014] nmol/L per year) with a relative decrease of −3.76 [−10.50 to 5.08] %.

**Table 3.** Relationship of baseline SHBG and free T and glucose metabolism parameters in MLWOH

	SHBG (nmol/L)		Free T (nmol/L)	
Outcome	Model 1	Model 2	Model 1	Model 2
β (*P*-value)				
Matsuda index	**0.147 (<.001)**	**0.124 (<.001)**	10.7 (.060)	1.63 (.766)
HOMA-IR	−**0.021 (.001)**	−0.005 (.392)	−2.38 (.061)	−0.770 (.544)
Disposition index	**0.194 (.001)**	0.119 (.224)	16.623 (.148)	−0.980 (.950)

Data expressed as beta (β) coefficients and (*P*-values). Model 1: Adjusted for baseline age and follow-up time. Model 2: Adjusted for baseline age; fat mass index; visceral adipose tissue area; smoking; follow-up time; and hypertension, diabetes, and dyslipidaemia medication.

Abbreviations: free T, free testosterone; HOMA-IR, homeostasis model assessment of insulin resistance; MLWOH, men living without HIV.

When comparing the MWLH to the MLWOH, SHBG and total T concentrations at baseline were higher in MLWH (*P* < .001 for both), but for total T this was only significant in model 1, whereas for SHBG the difference persisted even after adjusting for FMI. Free T did not differ by HIV status at baseline (Supplementary Table 1 [45]).

### Prediction of Dysglycaemia and Glucose Metabolism Parameters at Follow-up

#### MLWOH

**Figure 2.**
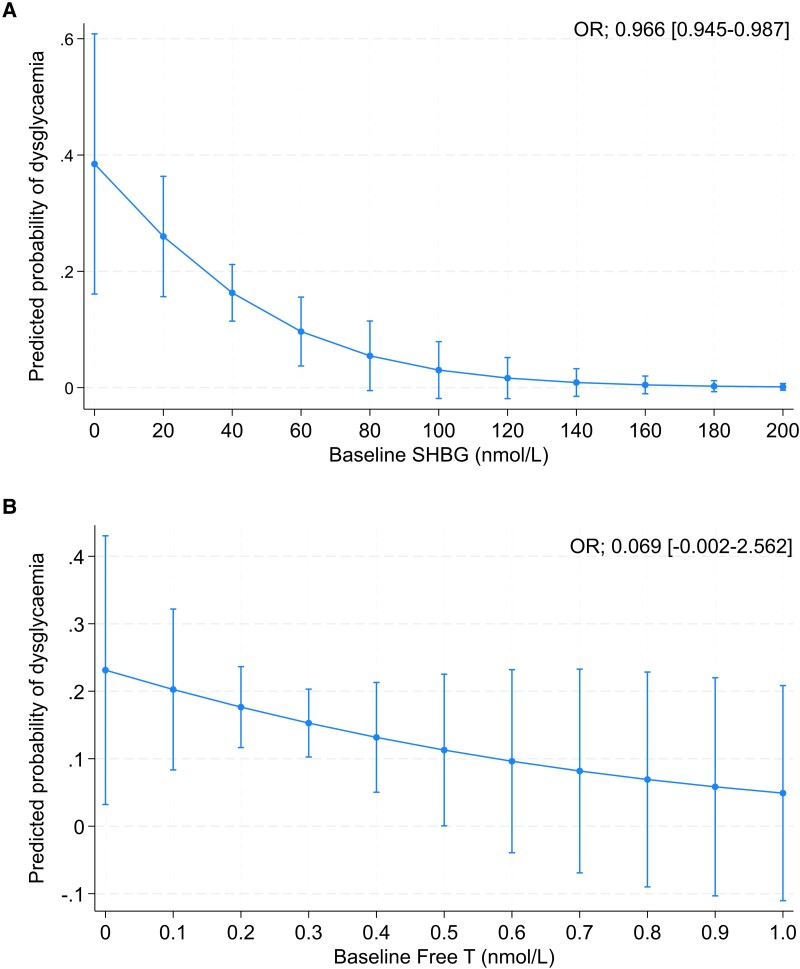
The association plots of baseline (A) SHBG and (B) free testosterone and incident dysglycaemia in middle-aged men living without HIV. Model adjusted for baseline age; follow-up time; fat mass index; visceral adipose tissue area; smoking; and hypertension, diabetes, and dyslipidaemia medication.

In MLWOH, the prevalence of dysglycaemia at follow-up was 17% (n = 55). Baseline SHBG was associated with a lower risk of incident dysglycaemia (OR [95% CI] 0.966 [0.945-0.987]) even after adjusting for chronic medication use and adiposity (Fig. 2A). Baseline free T was not associated with incident dysglycaemia at follow-up (0.069 [0.002-2.562], *P* = .147, Fig. 2B).

When exploring the associations between baseline SHBG levels and glucose metabolism parameters at follow-up, baseline SHBG was positively associated with insulin sensitivity also after adjusting for confounders and adiposity (Table 3). There was an inverse association between baseline SHBG and HOMA-IR and a positive association between baseline SHBG and DI, but these were no longer significant after adjustment for confounders (Table 3). Baseline free T was not associated with any parameters of glucose metabolism (Table 3).

#### MLWH

In MLWH, the prevalence of dysglycaemia at follow-up was 12% (n = 10). The prevalence of dysglycaemia did not differ by HIV status (*P* = .249). In contrast to MLWOH, baseline SHBG was not associated with the risk of incident dysglycaemia (OR [95% CI] 0.994 [0.971-1.02]), insulin sensitivity [β (*P*-value) 0.047 (0.134)], and HOMA-IR [−0.007 (0.136) or DI 0.142 (0.227) in MLWH. Free T was also not associated with incident dysglycaemia (OR [95% CI] 0.006 [−0.067-0.081]) or any parameters of glucose metabolism; insulin sensitivity [β (*P*-value) 6.92 (0.496)], HOMA-IR [−1.11 (0.445)], and DI [44.1 (0.245)] at follow-up in MWLH.

## Discussion

In a cohort of middle-aged Black African MLWOH, higher SHBG was associated with a lower risk of developing dysglycaemia ∼3 years later. SHBG was also associated with higher insulin sensitivity and β-cell function and inversely associated with insulin resistance ∼3 years later. Free T did not predict dysglycaemia and parameters of glucose metabolism. This is, to our knowledge, the first longitudinal study describing changes in SHBG and free T concentrations and their relationship with incident dysglycaemia in an African population. Compared to MLWOH, MLWH had higher SHBG levels that increased over time. Despite higher levels, SHBG was not associated with dysglycaemia and glucose metabolism parameters ∼3 years later.

SHBG has previously been regarded as a glycoprotein that exclusively transports sex hormones; however, recent studies have shown that it can independently elicit biological processes and is involved in cell signaling [46-48]. In this cohort of middle-aged Black African MLWOH, we showed for the first time that baseline SHBG was associated with a lower risk of incident dysglycaemia at 3 years follow-up. These findings are in line with several American and European cross-sectional [1] and longitudinal studies [3, 10, 49] that reported an association between higher SHBG and lower odds of T2D in middle-aged men. Mendelian randomization studies have linked 3 single nucleotide polymorphisms within the SHBG gene to the risk of developing T2D, suggesting that low SHBG may have a role in the pathogenesis of T2D [49, 50]. However, these studies included participants of predominantly White European ancestry, and these genetic variants only accounted for 2.2% of the variance in SHBG levels [49]. We also show for the first time in Black Africans that baseline SHBG was associated with greater insulin sensitivity and β-cell function at follow-up. This strengthens the growing body of evidence supporting the notion that SHBG confers a protective role in metabolic health and may be involved in the pathogenesis of T2D [51, 52]. However, the specific putative mechanisms of the association between SHBG on the one hand and insulin sensitivity and glucose metabolism on the other still needs to be investigated [51]. A decrease in SHBG has been suggested to decrease insulin sensitivity via direct mechanisms or activation of the SHBG receptor complex [53]. As hyperinsulinemia suppresses the production of SHBG, this could lead to a vicious circle leading to pronounced hyperinsulinemia via a compensatory increase in β-cell production of insulin [53]. This may contribute to the development of glucose intolerance and, ultimately, the onset of diabetes [53]. Further, it has been reported that SHBG plays a role in mitigating inflammation and accumulation of lipids in both macrophages and adipocytes. These functions could be pivotal in the protective capacity of SHBG, contributing to its ability to lower the risk of metabolic syndrome [54]. These findings overall underscore the potential significance of SHBG as a biomarker and implicate its role in modulating key aspects of glucose metabolism.

In our cohort of middle-aged Black African MLWOH, SHBG did not change over the 3-year follow-up period, whereas studies including healthy middle-aged Australian, European, and American men predominantly of European ancestry showed an increase in SHBG and a decrease in free T with age; however, their follow-up time was longer (ranging from 7 to 15 years) [55-57]. In contrast, we showed that free T did not change with time in the middle-aged Black African MLWOH population. Apart from the relatively short follow-up period (∼3 years), the absence of a decline in free T may also be attributed to the concurrent stability of SHBG, given that an elevation in SHBG is linked to a reduction in free T [58].

Unlike SHBG, our study showed no significant association between free T and dysglycaemia and glucose metabolism parameters. While some European and American studies have demonstrated associations between testosterone levels and dysglycaemia [59, 60], others have found no associations [1, 61]. The lack of consistent findings could also be attributed to several factors. First, variations in study populations, including demographic characteristics and genetic and environmental factors specific to the studied population, can influence outcomes. Moreover, the lack of consistent findings could also be attributed to variations in study methodologies, such as differing follow-up time, dysglycaemia criteria, and methods used to measure testosterone levels, warranting further research in this area. The precise impact of testosterone on β-cell function and glucose metabolism is not well understood [2]. From a physiological standpoint, testosterone acts on β-cells, augmenting glucose-stimulated insulin secretion [1, 2, 62, 63]. Conversely, testosterone deficiency in men is implicated in β-cell dysfunction and the attenuation of insulinotropic effects, potentially predisposing individuals to obesity and impaired glucose metabolism [2]. The current dearth of data concerning the metabolic implications of longitudinal changes in free T underscores the necessity for additional research in this area.

Interestingly in MLWH, SHBG concentrations were higher at baseline and increased with time compared to MLWOH. Most studies have reported an increase in SHBG with age in a general population of men, but few have focused on the effects of HIV [55, 56, 58]. This is consistent with a 15-year follow-up study of predominantly White American (82%) men living with HIV aged ≥45 years that showed an increase in SHBG with age [10]. The mechanisms underlying the higher concentration of SHBG in MLWH remain unclear, but it has been speculated that it may be a compensatory mechanism to decrease the systemic inflammation that persists during HIV infection [10]. In vitro studies have shown that SHBG decreases the production of cytokines through different pathways such as inhibiting the phosphorylation of the c-Jun N-terminal kinase and extracellular regulated kinases [64], increasing hepatocyte nuclear factor-4 alpha gene expression, or suppressing the expression of transcriptional factors such as the activator protein-1 [54, 65]. However, Dias et al concluded that the greater increase in SHBG with age in MLWH cannot be attributed to systemic inflammation. They suggested that an increase in N-linked glycosylation may be contributing to the slow clearance of SHBG from plasma [10], and this has been supported by in vivo studies [58].

Despite the elevated SHBG levels in MLWH, no significant associations were observed with incident dysglycaemia and insulin sensitivity. The prevalence of dysglycaemia also did not differ by HIV status (*P* = .249). This raises compelling questions about the translational impact of increased SHBG levels on the risk of T2D in people living with HIV. This may suggest that elevated SHBG levels do not necessarily translate to reduced T2D risk in MLWH. The reasons for the lack of association between SHBG and dysglycaemia in MLWH are not clear. However, we can speculate that due to the hyperbolic nature of the relationship between SHBG and the risk of dysglycaemia, increasing SHBG from already elevated SHBG levels may not confer reduced risk in MLWH. Additionally, our study is limited by the relatively small sample size of MLWH, as it had lower statistical power than the MLWOH cohort. Further studies are needed to elucidate the implications of high SHBG levels and diabetes risk in MLWH.

There have been contradictory reports as to whether the decline in free T differs by HIV status, with some studies reporting no differences in free T [6] and others a lower free T [1] and premature decline of total T in MLWH vs those without HIV [66]. Interestingly, in our study even though there was no decrease in free T in the MLWOH, free T levels declined significantly over the 3 years in MLWH. The reduction in free T in MLWH may be due to several factors including chronic inflammation, ART, body composition changes, and comorbidities [67] but also the increase in SHBG. We did not measure free T directly but rather used the Vermeulen equation, which is reliant on the SHBG levels; however, the Vermeulen equation is a widely accepted formula that has been validated against direct measurements of free T in different populations and has been used in numerous published studies [1, 6, 37, 68]. More research on the effect of HIV on the gonadal axis and direct measurement of free T is required.

This study adds to the body of information on SHBG and free T changes with age and their relationship with dysglycaemia. Notably, it is the first study to address longitudinal hormone changes in Black African men with and without HIV. It further supports the growing evidence that SHBG has independent and different effects from the sex hormones that it transports. Our study showed that 1-unit higher baseline SHBG level was associated with a 3.4% reduction in dysglycaemia risk at the ∼3-year follow-up. While this association is statistically significant, we acknowledge that the clinical significance of this association remains to be established. Rather than only focusing on total T as often reported in the literature, we calculated free T and explored the changes with age and its implications for T2D risk, also examining the influence of HIV infection in these relationships. However, we acknowledge that using calculated free T instead of directly measuring free T is a limitation. A longer follow-up time is also needed to establish these age-related changes and implications for T2D risk. A larger number of MLWH may also be needed for more power and generalizable results. We could not tease out the effects of the different antiretrovirals as 84% of those on treatment were on the same drug (non-nucleoside reverse transcriptase inhibitors). Further, we only had fasting glucose measurements at baseline and used them to exclude participants with impaired fasting glucose and T2D at baseline. Future studies could explore the comparative efficacy of glycemic control methods by incorporating both glycated hemoglobin and OGTT measurements and investigate their associations with SHBG and free T levels to allow a comprehensive evaluation of the effects of different glycemic control and sex hormones in middle-aged men.

In conclusion, we show for the first time that low SHBG levels predict the development of dysglycaemia in Black SA middle-aged MLWOH, whereas free T was not associated with dysglycaemia and measures of glucose metabolism. Despite higher SHBG in MLWH, we further show that SHBG and free T are not associated with dysglycaemia in Black African MLWH. Further studies with a larger sample of MLWH and a longer follow-up are needed to elucidate age-related changes in SHBG and androgens and the implications for T2D risk.

## Data Availability

Some or all datasets generated during and/or analyzed during the current study are not publicly available but are available from the corresponding author on reasonable request.
